# Building the empire: a multi specialist, multi centre pragmatic trial

**DOI:** 10.1186/1745-6215-14-S1-P88

**Published:** 2013-11-29

**Authors:** Rachel Rikunenko, Julie Dodds, Shakila Thangaratinam

**Affiliations:** 1 Women's Health Research Unit, Queen Mary University of London * on behalf of EMPIRE collaborative group, London, UK

## Background

There is a 10-fold increase in mortality among pregnant women with epilepsy, which greatly exceeds the two to three-fold rate of deaths observed in all people with epilepsy. Risks of uncontrolled convulsive seizures in the mother outweigh potential risk of the medication. The levels of anti epileptic drugs (AEDs) have been shown to reduce during pregnancy.

EMPiRE is a randomised controlled trial to determine whether Therapeutic Drug Monitoring reduces risk of seizure deterioration compared to Clinical Features monitoring alone. The trial aims to recruit 1,000 participants. We report on the challenges of recruitment.

## Challenges

We were faced with a number of challenges: 1) The number of eligible patients has been less than originally predicted. 2) There has been a lack of obstetric/neurology collaboration. 3) The levels of research experience/infrastructure varied with sites requiring additional support. 4) There was a lack of clinic awareness/late referrals making it difficult to capture patients.

## Solutions

We developed a number of strategies to boost recruitment. The site recruitment target increased from 15 to 44 and sites were encouraged to apply for service support funds. A focus was placed on recruiting enthusiastic investigators and monthly collaborator forums were initiated to identify difficulties and successes in recruitment and study conduct. Regional co-ordinating research midwives were employed to support sites. Epilepsy Action became involved in all stages of study development and conduct. We also disseminated monthly update newsletters and regional trial leads for neurology and obstetrics promoted the trial at a local level.

